# What motivates young physicians? – a qualitative analysis of the learning climate in specialist medical training

**DOI:** 10.1186/s12909-015-0461-8

**Published:** 2015-10-15

**Authors:** Peter Iblher, Marzellus Hofmann, Michaela Zupanic, Georg Breuer

**Affiliations:** 1 Institute for Teaching and Educational Research in Health Sciences, Witten/Herdecke University, Alfred-Herrhausen-Str. 50, 58448 Witten, Germany; 2 University of Lübeck Clinic for Anaesthesiology and Intensive Care Medicine, Ratzeburger Allee 160, 23538 Lübeck, Germany; 3 Student Dean’s Office, School of Medicine, Witten/Herdecke University, Alfred-Herrhausen-Str. 50, 58448 Witten, Germany; 4 Erlangen-Nürnberg University Clinic for Anaesthesiology, Krankenhausstr. 12, 91054 Erlangen, Germany

**Keywords:** Postgraduate medical training, Learning climate, Assessment, Feedback, Curriculum

## Abstract

**Background:**

Not least the much-invoked shortage of physicians in the current and the next generation has resulted in a wide range of efforts to improve postgraduate medical training. This is also in the focus of the current healthcare policy debate. Furthermore, quality and scope of available postgraduate training are important locational advantages in the competition for medical doctors. This study investigates the preferences and concerns that German house officers (HOs) have about their current postgraduate training. It also highlights how HOs evaluate the quality of their current postgraduate training and the learning environment.

**Methods:**

HOs were asked to answer the question: “Which things are of capital importance to you personally in your medical training?”, using a free text format. The survey was conducted web based (Lime survey) and all data was anonymized. Summarizing qualitative analyses were performed using the software tool MaxQDA.

**Results:**

A total of 255 HOs participated in this study (female: *n* = 129/50.6 %; male: *n* = 126/49.4 %; age: 32 + 6 years) associated with 17 different German hospitals and from four medical specialties. Ten categories were generated from a total of 366 free text answers: 1. methodology of learning (*n* = 66), 2. supervision (*n* = 66), 3. learning structure (*n* = 61), 4. teaching competence (*n* = 37), 5. dedication (*n* = 34), 6. work climate (*n* = 29), 7. feedback/communication (*n* = 22), 8. challenge/patient safety (*n* = 21), 9. time/resources (*n* = 17), 10. personal security/safety (*n* = 13).

**Conclusions:**

HOs want a reliable and curriculum-guided learning structure. Different studying techniques should be used with sufficient (time) resources available in a trusting and communicative learning environment. Competent and dedicated instructors are expected to give individual and specific feedback to the HOs on individual strengths and deficits. Instructors should develop educational concepts in cooperation with the HOs and at the same time avoid excessive demands on HOs or hazards to patients.

## Background

The education and postgraduate training of medical doctors poses a challenge for society in general and is in the focus of the healthcare policy debate. Not least the much-invoked shortage of physicians in the current and the next generation has resulted in a wide range of efforts to improve postgraduate medical training.

In some regions hospitals and doctors’ offices have been competing for medical professionals and qualified staff for some time. Many studies show that not only family orientation, work-life balance and financial remuneration constitute locational advantages in this competition but also the quality and scope of available postgraduate training [1]. Investments in improved advanced training appear therefore worthwhile in anticipation of future demographic change [2]. An appropriate learning environment in the clinical setting that permits high-quality inspirational education must be assumed to be a conditio sine qua non for successful training [3] and is reflected in a variety of aspects such as the quality of supervision, instructors, spatial conditions, and working and learning environments [4]. These aspects may be summed up under “learning climate” [5–7].

In Germany, specialty training is performed at approved specialty training institutions during appropriately remunerated, full-time practice of the medical profession. It is given under the direction of authorised physicians, in the form of practical activity and theoretical instruction, and partly through successful participation in courses recognised by the responsible State Chambers of Physicians in Germany (German Medical Association). In general, House Officers (HOs) have to work 5–6 years in the designated discipline. Specialty training must be documented, and its completion is assessed on the basis of the certificates issued by the authorised specialist trainers and an examination.

The main objective of this study is to investigate the preferences and concerns that German house officers (HOs) have about their learning. Furthermore, based on data collected, the study exemplarily highlights how HOs evaluate the quality of their current postgraduate training and the learning environment. This may contribute to a better understanding of problems in the process of postgraduate training and identify parameters for quality improvement.

## Methods

The study was submitted to the Ethics Committee at Witten/Herdecke University and did not meet with any ethical reservations concerning implementation. Particularly, the ethics committee approved not obtaining informed consent and authorized the study group that consent was taken as implied through action. All participants were aware that their responses were being used for a study and had the opportunity to refuse participation. They were clear that these responses were anonymous.

In the context of a German-language quantitative replication study on the D-RECT questionnaire (Dutch Residency Educational Climate Test) (Iblher et al. 2015, GMS Z Med Ausbild, in print) a free text item was included concerning a qualitative analysis of the learning climate in postgraduate medical education:

“Name an issue which you personally believe is the most important one in postgraduate education. You may also add comments on your postgraduate training, the questionnaire or general issues.”

The survey was conducted online (Lime Survey). To get a representative sample of German House officers, 17 hospitals out of 6/16 German federal states were included (Northern, Middle and Southern Germany) as potential participants. The Head of the selected departments and local house officers’ representatives were contacted, and after consent the authors received email-addresses of potential participants (PP). Overall, 324 HOs were contacted via email with the link to the questionnaire and simultaneously a link to refuse participation. After two weeks a reminder was sent to non-responders automatically and again after another two weeks. PPs who did not respond after another two weeks were excluded. Evaluation of collected data was anonymized. Summarizing qualitative content analyses [8] were performed using the software tool MaxQDA for qualitative data analysis.

## Results

From a total of 324 PPs, 19 were excluded as they had already passed their Board certification as medical specialists, 8 PPs were on maternity leave and did not receive the link, 22 PPs rejected the invitation, and 20 PPs did not answer at all. Therefore, the sample was compiled from 255 HOs (female: *n* = 129/ 50.6 %; male: *n* = 126/ 49.4 %) associated with 17 different German hospitals (Table 1) and four medical specialties (Table 2). The average age of participants was 32 ± 6 years. Table 3 shows the distribution for years of postgraduate training. During the study the authors followed the summative qualitative text analysis approach [8]. A total of 366 free text answers were registered and in a first level content analysis assigned to 22 different categories (PI) using the software MaxQDA. Data was displayed in a tabular summary including quantitative results calculated by MaxQDA at the end of every step (number of text segments/ percentage per category). In a second step the authors (MZ, GB) analyzed the generated category system, identified instances of overlapping and reduced the number of categories to 15. A third step with renewed identification and aggregation of categories (PI) with similar or identical meaning finally yielded 10 categories to which the 366 free text items were assigned for the purposes of rudimentary quantification. At the end, all authors (PI, MH, MZ, GB) discussed and approved the final version.

**Table 1:** Breakdown of respondents according to states and locations. (*n* = 2: no details provided)

German state	Number of locations	Number of respondents
Schleswig-Holstein	6	104
Mecklenburg-Western Pomerania	3	39
North Rhine-Westphalia	3	33
Berlin	2	40
Bavaria	1	15
Hamburg	2	22
total	17	253

**Table 2:** Breakdown of respondents according to discipline

Discipline	Frequency	Percentage
Anesthesiology	198	77.6
Internal medicine	36	14.1
Pediatric surgery	13	5.1
General medicine	8	3.1

**Table 3:** Breakdown of respondents according to level/year of postgraduate training

Year of postgraduate training	Frequency	Percentage
1^st^ – 2^nd^	93	36.5
3^rd^ – 4^th^	80	29.8
5^th^ – 6^th^	52	20.4
7^th^ – 9^th^	23	9.0
>9^th^ year	10	4.0

**Fig. 1:**
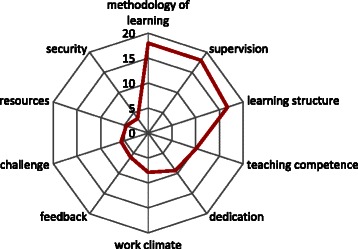
Percentage frequencies of 366 responses in ten categories

The ten categories are described below, in descending order on the basis of individual entries (*n* = number of individual entries in the respective category, cf. Fig. 1, Table 4). Quotations of HOs are written quote unquote:

**Table 4:** Categories with examples

Category	Definition	Examples
1. Methodology of learning	training methods which might be made available for instruction	-“joint deliberation on case histories”
		-“learning from practical cases”
		-“practice – many cases – exotic procedures”
2. Mentoring and supervision	personal and continuous guidance and supervision and the reliable availability of a personal contact	-“A personal postgraduate training commissary would be nice”,
		-“a contact such as a senior physician who looks after me and gives specific and targeted instruction”
		-“personal mentoring”
3. Structured postgraduate training	Curricular structures, transparency and reliability in planing	-“a structured curriculum that /…/ is actually put into practice”
		-“reliability in the planning of advanced training and adherence to agreements”;
		-“not to be disadvantaged as a woman”
4. Teaching competences	subject-specific and didactic-methodological competences of instructing physicians	-“introduction and guidance by experienced physicians so that I can learn from experience”
		-“being introduced to new activities by senior physicians and not by newly instructed assistants without experience”
		“conveyance of knowledge and skills”
5. Dedication and readiness to teach	the implicit and dedicated readiness to teach on the part of superiors	-“not having to beg for instruction all the time”,
		-“that chief and senior physicians act as role models, give us encouragement, and are good instructors”,
		“dedicated superiors”,
6. Work climate	culture of interaction in the working environment	-“respectful cooperation”
		-“with the option to make mistakes, discuss them and learn from them”
7. Feedback/Communication	Feedback culture	-“constructive feedback”
		-“target vs. actual control”
		-“lack of criticism is not a substitute for praise nor for an interview on educational attainment”
8. Challenge/patient safety	the right balance of challenge and overload during training	-“individual responsibility”
		-“responsibility without being left alone”
		-“learning by doing means much mental stress”
9. Time/resources	Time pressure due to daily structural demands	-“most specialists and senior physicians have no time for thorough practical instruction, being too much involved in their areas of responsibility”
		-“time!!!… and no pressure to meet corporate targets”
10. Personal security and confidence	Safety requirements	-“being acquainted or monitored well in order to gain security/confidence to meet growing demands, without being overwhelmed and therefore making mistakes”

1. Methodology of learning (*n* = 66)
  The focus in this category is on the postgraduate training methods mentioned by the HOs which might be made available for instruction. In general, interviewees wanted regular advanced training units and instruction (during working hours). Beside case conferences (“discussion”, “joint deliberation on case histories”, “learning from practical cases”), HOs specifically expressed the wish for practical exercises, also using phantoms and simulators: (“practical exercises”, “acquisition of specific manual skills”, “training in operative techniques”, “not just theory but training in functional diagnostics”, and “practice – many cases – exotic procedures”).
2. Mentoring and supervision (*n* = 66)
  This category concerns the wish of HOs for personal and continuous guidance and supervision and the reliable availability of a personal contact: (“A personal postgraduate training commissary would be nice”, “a contact such as a senior physician who looks after me and gives specific and targeted instruction”, “personal mentoring”).
3. Structured postgraduate training (*n* = 61)
  This category covers curricular structures in postgraduate training, transparency and reliability in planning. The free texts repeatedly point out a lack, or lacking implementation of, defined training structures which HOs would prefer: (“There is no structured training, no rotation schedule, no instructors designated as such…”, “no structured teaching, only learning by doing”, (a wish for) “a structured curriculum that does not only exist on paper but is actually put into practice”). The HOs report that no mandatory training contents are conveyed: (“Structured postgraduate training as stipulated by the medical association does not take place”, (it is hard to) “acquire actual practical experience that is not only attested in writing and corresponds to pertinent regulations for postgraduate medical training”); learning objectives are not clearly communicated: (“I was/am insecure as to how postgraduate training should be organized”). In general, HOs believe reliability and transparency to be important elements of postgraduate training: (“reliability in the planning of advanced training and adherence to agreements”; “the objectives for postgraduate training which were defined in target discussions are implemented insufficiently … (I feel) some degree of resignation,” “neglected standards/checklists”, “not to be disadvantaged as a woman”).
4. Teaching competences (*n* = 37)
  The focus in this category is on the subject-specific and didactic-methodological competences of instructing physicians. For the HOs it is important to receive advanced training from experienced specialists and senior physicians (“introduction and guidance by experienced physicians so that I can learn from (their) experience”, “being introduced to new activities by senior physicians and not by newly instructed assistants without experience”) and from professional instructors (“professionality”) with didactic competences (“conveyance of knowledge and skills”).
5. Dedication and readiness to teach (*n* = 34)
  This category addresses the implicit and dedicated readiness to teach on the part of superiors (“not having to beg for instruction all the time”, “that chief and senior physicians act as role models, give us encouragement, and are good instructors”, “dedicated superiors”, “good teachers”) and their competences to motive the HO (“motivating support by mentor”). A key point of criticism is a lack of self-motivation among instructors (“no reason why training could not be considerably improved, but this fails despite continuous efforts because specialists and senior physicians are not motivated”, “… in my opinion, senior physicians who cultivate inactivity are absolutely unsuitable as role models!”).
6. Work climate (*n* = 29)
  This category concerns the culture of interaction in the working environment. The interviewees mentioned mutual respect (“respectful cooperation”), appreciation and recognition as well as a pleasant work climate with an open error handling culture (“with the option to make mistakes, discuss them and learn from them”). Some free texts underline the existence of such a team culture as a positive point (“the climate is alright despite all deficits in postgraduate training; no reason to be afraid of senior physicians and superiors”, “excellent team work among assistant physicians”, “basically the climate is very positive and free from anxiety”).
7. Feedback / Communication (n = 22)
  In this category the HOs frequently wrote only “feedback” as free text, sometimes specified as “constructive feedback”, “target vs. actual control”, “honest feedback”, “personal feedback”, “regular feedback”. Some free texts criticized a lack of intra-departmental feedback (“so far no reflection on my performance from my “own” senior physicians, only criticism (positive and negative) from other departments”, “no regular feedback in my postgraduate training”, “…but feedback on personal progress is being neglected”). Interviews on postgraduate training were another point of criticism in the free text answers (“no interviews on progress in postgraduate training”, “lack of criticism is not a substitute for praise nor for an interview on educational attainment”, “a structured interview on competences and possibly on learning objectives, scheduled before, during and after rotation, would be helpful and make sense”).
8. Challenge/ patient safety (n = 21)
  The focus in this category is on the right balance of challenge and overload during postgraduate training. HOs voice a wish for autonomous action (“individual responsibility”) but with professional support (“responsible and autonomous work”, “responsibility corresponding to the level of training, responsibility without being left alone”). Several interviewees explicitly reject and criticize the learning by doing approach (“learning by doing means much mental stress”) and express a wish for safety in patient care (“good patient care, i.e. sufficient supervision and feedback … and professional guidance”).
9. Time / resources (*n* = 17)
  HOs perceive training conditions in this category as in competition or even in conflict with daily structural demands of patient care (“it is inconvenient if staffing levels and pressures to meet the surgery target figures do not leave enough room for postgraduate training”, “most specialists and senior physicians have no time for thorough practical instruction, being too much involved in their areas of responsibility”), of available resources (“there is not enough time and opportunity to discuss and practice certain skills … no specialist or senior physician available all the time”, “just not enough expert personnel for a comprehensive training of all physicians in postgraduate training”) and of agreed targets (“time!!!… and no pressure to meet corporate targets”). Time to familiarize oneself with a ward is often too short (“time for introduction often too short or non-existent, rotations too short…, which makes sound learning/routines difficult”). There is also the wish to spend more time on patient care (“time to address the serious problems of some patients comprehensively…”).
10. Personal security and confidence (*n* = 13)
  This category constitutes an interface between the other categories, since security and confidence is addressed in free texts all the time but interviewees mention consecutive measures from other categories (“being acquainted or monitored well in order to gain security/confidence to meet growing demands, without being overwhelmed and therefore making mistakes”, “defined structures at the start that provide a certain framework and security…”). Personal confidence appears to play a larger role specifically at the start of postgraduate training (“At the beginning it is not easy to find your way in the complex clinical setting and your role or place. Quite often you feel lonely, unprepared and helpless”).

## Discussion

A qualitative content analysis was chosen to identify free and spontaneous assessments on the part of HOs. In conclusion, HOs primarily want reliable learning structures with practice orientation and curricular basis including various teaching methods that are suitable for professional practice (regular thematic advanced training, discussions, practical exercises) in a pleasant, trusting and communicative learning environment (work climate). Sufficient (time) resources should be made available for these purposes. The focus is on competent and dedicated individual supervision of HOs by specialists, chief and senior physicians or mentors, the aim being to enable and encourage HOs to work autonomously under supervision. Mentors should give targeted feedback to HOs on their personal strengths and deficits and on this basis should develop individual concepts for advanced training together with each HO, without overtaxing him or her or endangering patient safety.

In general, a clearly defined and realistic curriculum for postgraduate training serves to structure the training process and provides more transparency with regard to learning objectives. In particular, a staggered training schedule (e.g. according to trainee years) might help to clarify educational objectives to be achieved within a specific trainee year and to be verified. The consistent use of competence-based curricula which today can rightly be described as “state of the art” would therefore meet key claims made by HOs in this study. In this context, the German Medical Association’s initiatives for a new, competence-based model statute of specialist training must be seen as an important step towards establishing such transparent and practice-oriented curricula [9]. Curricula are required to allow for an adequate examination of HOs on the basis of clearly defined learning objectives and competences. On the one hand, this permits instructors to reach a realistic assessment of HOs that corresponds to the actual level of training. On the other, it corresponds to the wish for more feedback expressed by HOs in the sense of a formative and unsanctioned feedback with a focus on learning progress. It should be emphasized that adequate feedback follows defined rules which a user needs to assimilate [10] and, properly applied, have a significant impact on a HO’s learning processes [11]. Apart from the classical oral feedback in postgraduate training interviews, the term “workplace-based assessments” (WPBA) subsumes a range of examination tools which correspond to the wishes expressed by HOs for a structured feedback process in terms of methodology and content, and necessarily permit the assessment of practical competences at the highest competence level [12–14]. The following three categories are primarily assessed in a WPBA: 1. Observation of clinical activities (mini-clinical evaluation exercise / Mini-CEX; direct observation of procedural skills / DOPS), 2. Discussion of clinical cases (chart stimulated recall; case based discussion) and 3. Feedback from colleagues and patients (Multisource Feedback; 360° Feedback) [15].

The routine application of such techniques would affect and substantially improve many areas which HOs believe to be important according to this study, such as the wish for supervision with more and more direct feedback, a structured evaluation of competences and the application of supervised practical teaching scenarios. In this context the existence of a respectful and considerate communicative and team culture is an essential component of a positive learning environment which HOs are justified to claim in their postgraduate training [16].

Apart from the availability of time-related and human resources, the dedicated and competent training of HOs requires that instructors have acquired at least the fundamentals of medical pedagogy in addition to their subject-specific expertise. This should be made a requirement since qualification in this field would very likely have an immediate positive impact on learning competences and methodology which HOs named as important elements of good postgraduate training. Various training scenarios have been established which range from selective train-the-trainer courses to structured and comprehensive concepts of qualification in medical pedagogy [17]. The question how to influence dedication and motivation in instructors positively is by no means trivial since these aspects are influenced by a multitude of factors. But a minimum demand should be that instructors are given sufficient time for their teaching activities and that these activities should be properly appreciated [18]. By no means should instructors experience sanctions or disadvantages as a consequence of their teaching activities.

A critical and limiting factor is that the collective of respondents in this study comprised only four specialist disciplines with a proportional emphasis on anesthesiology and internal medicine. It would be interesting to include other disciplines in subsequent studies. The “voluntary response bias”, always to be considered in voluntary survey, should also be mentioned here, whereby the presentation in a system of categories serves to strengthen the representativeness of findings.

Based on the results of this study, consideration should be given to professional measures, which reflect not only the wishes expressed by HOs as described above but also latest findings from educational research. All parties concerned would greatly benefit from such measures in every respect: patients, those in charge of training, hospital operators and of course the HOs themselves.

## Conclusions

HOs want a reliable and curriculum-guided learning structure. Different studying techniques should be used with sufficient (time) resources available in a trusting and communicative learning environment. Competent and dedicated instructors are expected to give individual and specific feedback to the HOs on individual strengths and deficits. Instructors should develop educational concepts in cooperation with the HOs and at the same time avoid excessive demands on HOs or hazards to patients (Table 5).

**Table 5:** Portfolio of good postgraduate training for physicians (WPBA: workplace based assessment)

• Individually reliable and transparent curriculum
• Adequate range of learning methods with sufficient (time) resources
• Pleasant, trusting and communicative learning environment
• Competent and dedicated supervision with individual feedback (WPBA)
• Avoidance of overload and risks to patient safety
